# Polystyrene Upcycling via Photocatalytic and Non-Photocatalytic Degradation

**DOI:** 10.3390/molecules30153165

**Published:** 2025-07-29

**Authors:** Terry Yang, Yalan Xing

**Affiliations:** Department of Chemistry, Hofstra University, Hempstead, NY 11550, USA

**Keywords:** polystyrene, upcycling, photocatalytic, degradation, plastic

## Abstract

The rapid increase in polystyrene (PS) production has led to substantial growth in plastic waste, posing serious environmental and waste management challenges. Current disposal techniques are unsustainable, relying heavily on harsh conditions, high energy input, and generating environmentally harmful byproducts. This review critically discusses alternative green approaches for PS treatment through photocatalytic and non-photocatalytic upcycling methods. Photocatalytic methods utilize light energy (UV, visible, or broad-spectrum irradiation) to initiate radical reactions that cleave the inert carbon backbone of PS. In contrast, non-photocatalytic strategies achieve backbone degradation without direct light activation, often employing catalysts and thermal energy. Both approaches effectively transform PS waste into higher-value compounds, such as benzoic acid and acetophenone, though yields remain moderate for most reported methods. Current limitations, including catalyst performance, low yields, and impurities in real-world PS waste, are highlighted. Future directions toward enhancing the efficiency, selectivity, and scalability of PS upcycling processes are proposed to address the growing plastic waste crisis sustainably.

## 1. Introduction

Polystyrene (PS) has become a critical material across numerous industries due to its advantageous properties, including chemical inertness, light weight, and long durability. However, these same characteristics also contribute to an escalating environmental crisis [1]. PS is highly resistant to degradation and can persist in the environment for centuries [2]. Styrofoam, a common form of extruded PS, poses additional challenges due to its high volume-to-mass ratio, which diminishes recycling efficiency and economic viability [3,4]. With global plastic production exceeding 26 million tons annually and projected to rise further, plastic waste management has become an urgent global concern [5]. Disposal methods such as landfilling and incineration are energy-intensive, environmentally hazardous, and often economically unfeasible [6]. Mechanical recycling, while widely implemented, frequently results in downcycled materials with diminished properties, ultimately destined for landfill [7]. In contrast, chemical recycling aims to depolymerize plastics into monomers or valuable small molecules, though conventional PS recycling techniques (such as thermal or catalytic pyrolysis) often require harsh conditions, high energy input, and suffer from low product selectivity [8,9].

To overcome these limitations, upcycling has emerged as a promising strategy for plastic waste valorization. Unlike traditional recycling, upcycling converts waste materials into products of higher value, rather than simply restoring them to their original form [10]. In the case of PS, this can involve generating useful chemicals such as benzoic acid, acetophenone, and other small aromatics, offering both environmental and economic incentives. Many upcycling approaches also operate under mild reaction conditions, reducing energy demands and avoiding issues such as coking or polyaromatic formation commonly observed in high-temperature methods.

Several recent reviews have begun to address the current state of PS degradation and upcycling. Gautam et al. provided a comprehensive overview of PS degradation techniques, including both mechanical and chemical strategies, with an emphasis on sustainable solutions [11]. Ran et al. surveyed photocatalytic plastic upcycling, highlighting advances in catalyst design and light activation mechanisms for a broad range of polymers [12]. Likewise, Kokotos et al. reviewed photochemical approaches to plastic upcycling and recycling, identifying key achievements and future directions for visible-light-driven plastic conversion [13].

Despite these advances, the chemical upcycling of PS remains particularly challenging due to the lack of functional groups or backbone heteroatoms (unlike PET (polyethylene terephthalate) or polyesters), which makes activation and depolymerization more difficult [14]. Moreover, real-world PS waste often contains additives, dyes, or food contaminants that complicate reaction outcomes and catalyst compatibility that can result in reduced yields and purity [15].

This review aims to expand upon the recent literature by specifically focusing on the latest methods for PS upcycling via two broad categories:

(1) Photocatalytic upcycling, categorized by light source: UV-induced, visible-light-induced, and broad-spectrum/simulated sunlight; (2) non-photocatalytic upcycling, which includes thermal, oxidative, and catalytic strategies operating independently of light. Together, these approaches provide new opportunities for transforming PS waste into valuable chemical feedstocks, contributing to the vision of a circular plastic economy.

## 2. Photocatalytic Upcycling of PS

Photocatalytic upcycling of PS is a light-driven process that degrades the polymer into simpler and potentially high-value products. This approach offers an efficient and environmentally friendly route for PS degradation. Upon light irradiation, radicals are generated along the PS backbone, which can be directly oxidized by atmospheric oxygen to form peroxy radicals. These intermediates ultimately lead to cleavage of the polymer chains [16,17,18]. As a result, most photocatalytic upcycling methods discussed in this review are performed under an oxygen atmosphere to facilitate the formation of these reactive species. To enhance the degradation rate, photoinitiators and photocatalysts, such as peroxides, benzophenones, quinones, and metal complexes, are commonly employed to promote oxidative cleavage of the polymer backbone [19,20,21].

While these strategies have shown significant promise, achieving selective conversion of PS into a single product, such as benzoic acid, remains a challenge [22,23]. This section highlights recent advances in PS upcycling that utilize UV, visible, and broad-spectrum light sources in the presence of photocatalysts to generate valuable chemical products.

### 2.1. Visible-Light-Induced Photocatalytic Upcycling

**Scheme 1:**
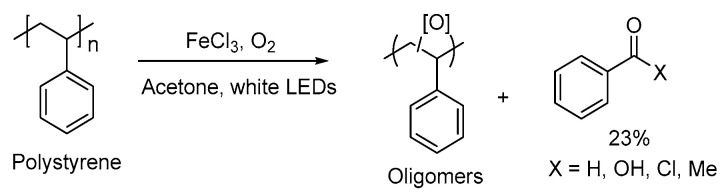
Photocatalytic degradation of PS via catalyst-controlled photo-oxidation using FeCl_3_.

In 2022, Sewon Oh and Erin E. Stache published an article involving a photocatalytic degradation reaction that used iron (III) chloride as the photocatalyst and white LEDs as the light sources (Scheme 1) [24]. This photochemical oxidative reaction primarily generated high-value benzoyl derivatives. Drawing from prior research, the authors noted that using hydrogen atom transfer (HAT) for photo-oxidative degradation enabled the generation of reactive species under visible light, avoiding the need for high-energy UV radiation and allowing for more selective control during hydrogen abstraction [19,20,25]. The researchers hypothesized that irradiation of FeCl_3_ with white LED light would generate chlorine radicals [26,27]. These radicals would then abstract benzylic C–H bonds from the PS backbone, initiating oxidation to form peroxyl radicals capable of promoting C–C bond cleavage.

**Scheme 2:**
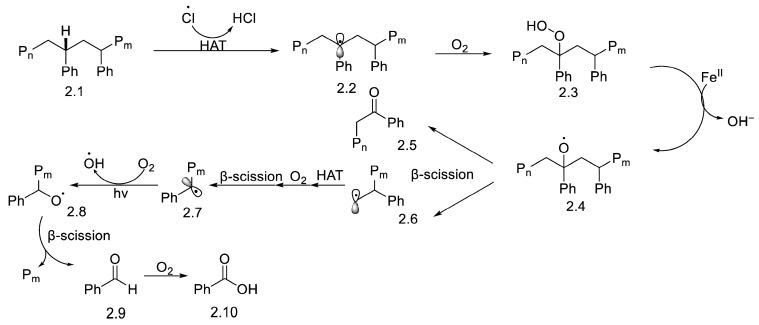
Mechanism of PS degradation via catalyst-controlled photo-oxidation using FeCl_3_.

As shown in Scheme 2, irradiation of FeCl_3_ with white LED light generated chlorine radicals which then abstracted electron-rich hydrogens from the polymer backbone to form radical 2.2. The resulting polymer radical was directly oxidized by atmospheric oxygen to form peroxide 2.3, leading to β-scission and polymer chain cleavage, yielding a phenyl ketone chain end (2.5) and a primary radical (2.6). Primary radical 2.6 subsequently underwent a series of oxidation and β-scission steps, generating benzaldehyde 2.9. Finally, atmospheric oxygen oxidized benzaldehyde to form the high-value product benzoic acid (2.10).

The reaction conditions in the study are mild, as the process is conducted at ambient temperature and atmospheric pressure without the use of harmful or expensive chemicals, indicating potential cost-effectiveness and environmental compatibility. The method was successfully scaled up to 20 g and tested on various commercial PS products, including a black coffee lid, a clear container lid, Styrofoam, and a Styrofoam coffee lid containing additional composite and insoluble materials. All commercial samples were effectively degraded except for the black coffee lid, which showed a low product yield due to the presence of black dye that inhibited light penetration. Notably, this method was conceptually scaled on the gram scale using flow chemistry, but no large-scale continuous flow implementation was reported. Nonetheless, the simplicity of the reagents and conditions suggests that this method holds promise for future economical scale-up.

**Scheme 3:**
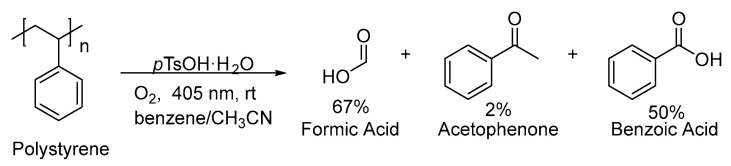
Photocatalytic degradation of PS via oxidative cleavage of PS by singlet oxygen in presence of *p*TsOH·H_2_O.

In 2022, Huang and coworkers published a study describing a novel photocatalytic degradation reaction using singlet oxygen (^1^O_2_), a well-known reactive oxygen species (ROS), to initiate oxidation of PS (Scheme 3) [28]. The reaction employed *p*TsOH·H_2_O and visible light at a wavelength of 405 nm as the light source. The primary products formed were benzoic acid, acetophenone, and formic acid at yields of 50%, 2%, and 67%, respectively. Since the singlet oxygen possesses a relatively high energy (94 kJ/mol) compared to the ground-state molecule (^3^O_2_), the researchers hypothesized that ^1^O_2_ can oxidize various organic molecules independent of temperature. Specifically, they proposed that ^1^O_2_ could abstract hydrogen from the weak tertiary benzylic C–H bond in PS, thereby initiating its chemical degradation [29]. The choice of solvent was found to significantly influence product formation. When solvents such as benzene, acetonitrile (CH_3_CN), acetone, or ethyl acetate (EtOAc) were used, only trace or no amounts of target products were obtained. In contrast, dichloroethane (DCE) produced moderate yields. Optimal results were achieved when *p*TsOH·H_2_O was used as the catalyst and benzene served as the solvent. However, benzene could be substituted with DCE or EtOAc, albeit with slightly reduced yields.

**Scheme 4:**
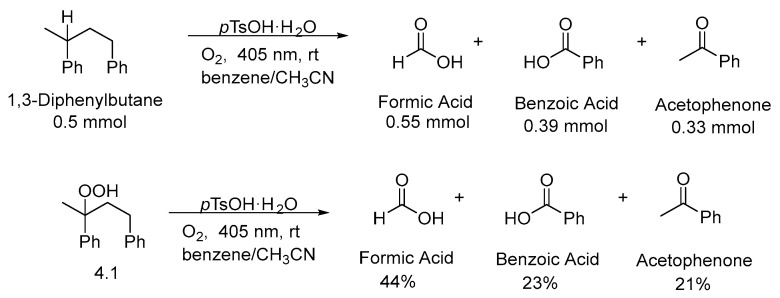
Oxidative cleavage pathways of 1,3-diphenylbutane in presence of *p*TsOH·H_2_O.

To gain deeper insight into the reaction mechanism, 1,3-diphenylbutane (0.5 mmol) was utilized as a model substrate and subjected to the standard oxidation conditions (Scheme 4). The reaction produced formic acid (0.55 mmol), benzoic acid (0.39 mmol), and acetophenone (0.33 mmol). Computational analysis suggested the formation of peroxide 4.1 via oxidation by singlet oxygen. This hypothesis was supported by experimental studies. When peroxide 4.1 was subjected to the standard oxidation conditions, formic acid, benzoic acid, and acetophenone were again observed.

The reaction conditions are mild, conducted at ambient temperature and pressure without the use of toxic or expensive chemicals, suggesting the method is both environmentally friendly and potentially cost-effective. When PS was treated under standard conditions in the presence of DPA or NaN_3_ (^1^O_2_ quenchers) or in the absence of light, no conversion or expected degradation products were observed. These results indicate that both ^1^O_2_ and light are essential for the reaction. Various PS waste materials, including cup lids, yogurt containers, loose-fill chips, EPS foam, food boxes, and lab weighing boats, were successfully degraded under these conditions. This method has been conceptually scaled to the gram level using flow chemistry, supporting its potential for future process intensification and scale-up.

**Scheme 5:**
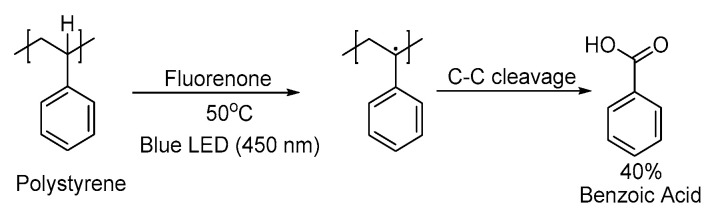
Photocatalytic degradation of PS catalyzed by fluorenone.

**Scheme 6:**
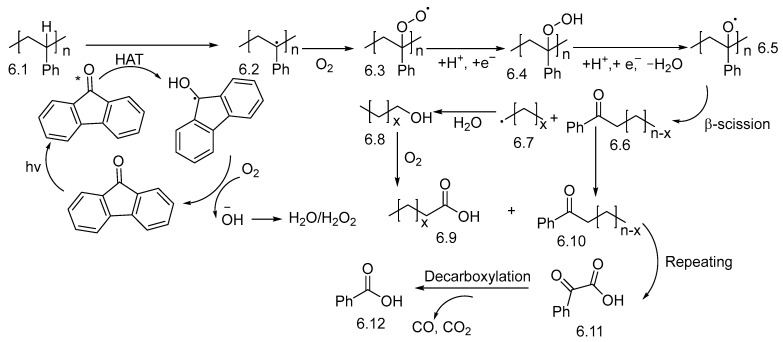
In-depth mechanism of photocatalysis and oxidation pathway catalyzed by fluorenone. * meant the excited state of the fluorenone.

In 2022, Tengfei Li and coworkers developed a photocatalytic degradation method using fluorenone as the photocatalyst and a blue LED (450 nm) as the light source (Scheme 5) [30]. Benzoic acid was obtained as the primary product at 40% yield. The reaction is proposed to proceed via a C−H bond oxidation pathway involving HAT, as supported by computational studies (Scheme 6). Upon blue-light irradiation [31], fluorenone is excited to its active state, initiating a HAT process between PS 6.1 and the excited fluorenone to form radical 6.2. This radical is subsequently oxygenated to yield peroxyl radical 6.3. Further HAT by the peroxyl radical produces intermediate 6.4, and elimination of H_2_O gives intermediate 6.5. This is followed by β-scission, oxidation, and decarboxylation, ultimately furnishing benzoic acid (6.12) as the final product.

The reaction conditions are mild, proceeding at ambient temperatures and atmospheric pressure without the use of toxic or expensive catalysts, which contributes to the method’s environmental and economic viability. The fluorenone catalyst can be easily regenerated by oxygen gas with an efficiency of 95%, reducing both the cost and the amount of material required per run. Both pure PS and Styrofoam were tested, and in both cases successful degradation was achieved. This method has also been successfully demonstrated at an industrially relevant scale through gram-scale deconstruction of both pure PS and Styrofoam.

### 2.2. UV-Light-Induced Photocatalytic Upcycling

**Scheme 7:**
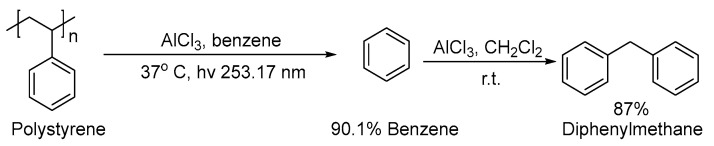
Photocatalytic degradation of PS via cascade degradation catalyzed by AlCl_3_.

In 2022, Zhen Xu and coworkers published a study on the photocatalytic degradation of PS using aluminum (III) chloride as the photocatalyst and UV light at 253.17 nm as the light source (Scheme 7) [32]. This reaction photochemically generated a mixture of light and heavy phases with benzene as the majority intermediate, formed through a Fridel–Crafts reaction. The light phase accounted for 88.4% of the mixture and consisted of benzene along with byproducts such as ethyl benzene, cumene, tert-butylbenzene, indane, 1-methylindane, 1-methylindene, diphenylmethane (DMP), phenylindene, and methyl phenylindene. The heavy phase, comprising 10.7%, contained inorganic salts and organics, including indanes, indenes, and heavy aromatics. The mixture was subsequently valorized with dichloromethane to form high-value DPM in 87% yield. DPM can further serve as a precursor for the synthesis of other valuable compounds such as benzophenone, 1,2-diphenylethane, and related derivatives.

This transformation proceeds under mild reaction conditions, conducted at ambient temperature and atmospheric pressure, and avoids the usage of harmful or expensive chemicals. This method was scaled up to 10 g and 1 kg using raw waste PS in the laboratory and resulted in successful degradation for both scales. A techno-economic analysis was also performed for a 100-metric-ton scale-up, demonstrating high profitability and low sensitivity to market fluctuations, supporting its potential for industrial application.

**Scheme 8:**
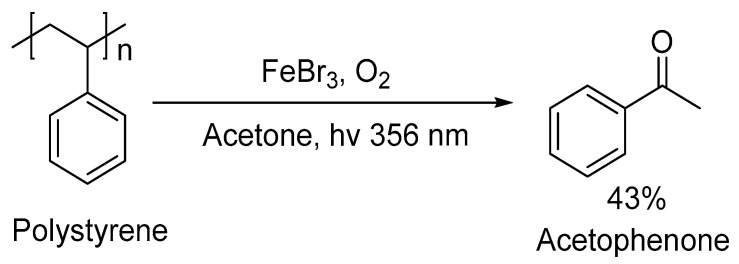
Photocatalytic degradation of PS via C-H bond abstraction by FeBr_3_.

**Scheme 9:**
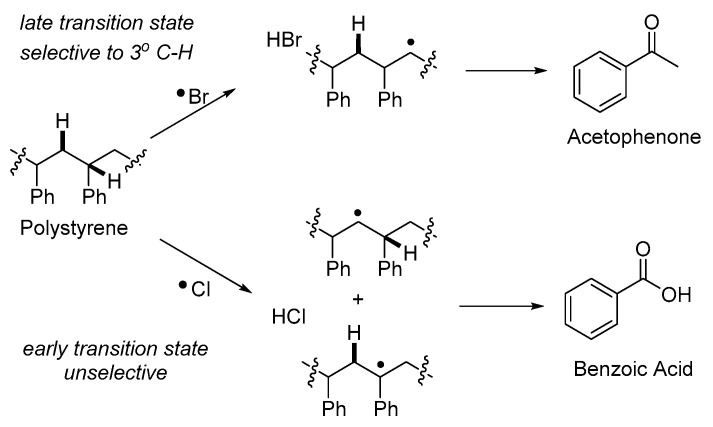
Mechanism of PS photo-oxidative degradation by two halogen radical protocols.

In 2023, Sewon Oh and Erin E. Stache published a follow-up study on photocatalytic degradation of PS, building on their earlier work [24]. In this study, instead of using iron (III) chloride (FeCl_3_) as the photocatalyst, they used iron (III) bromide (FeBr_3_) and a UV light source at 356 nm (Scheme 8) [33]. The reaction yielded acetophenone as the primary product, instead of benzoic acid, with a 43% yield. Through comprehensive mechanistic studies, the authors proposed divergent PS degradation pathways involving HAT mechanisms for FeCl_3_ and FeBr_3_. The key difference between the two pathways lies in the bond dissociation energy (BDE) of the hydrogen halides formed (Scheme 9). The BDE of H–Cl is 103 kcal/mol, leading to an early transition state and non-selective C–H abstraction [34]. In contrast, the BDE of H–Br is 87 kcal/mol, which results in a later transition state and greater selectivity for tertiary (3°) C–H bonds [35,36]. The authors also conducted small-molecule model studies to mimic the polymer chain ends, discovering that the presence of methyl groups is essential for the formation of acetophenone.

It was found that PS chains lacking methyl groups at both chain ends produced significantly less acetophenone compared to chains with methyl groups at both ends. This finding was further supported by tests on commercial PS products, which contained different structural variants of PS and resulted in varying yields of benzoic acid and acetophenone. The authors envision that this improved understanding of the PS degradation mechanism will aid in the development of optimized conditions for selectively producing either benzoic acid or acetophenone in higher yields and at larger scales. Improved selectivity and yield optimization not only enhance the value of the degradation products but also increase the economic viability of the process, especially if targeted product recovery can be efficiently achieved in larger-scale operations.

**Scheme 10:**
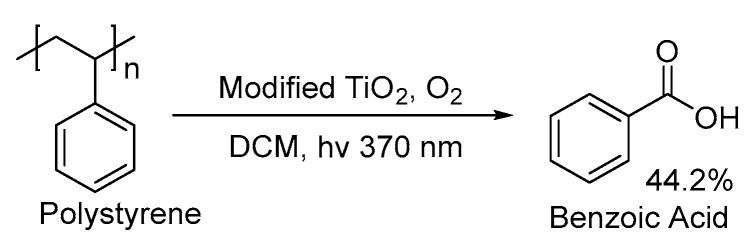
Photocatalytic degradation of PS catalyzed by modified TiO_2_.

**Scheme 11:**
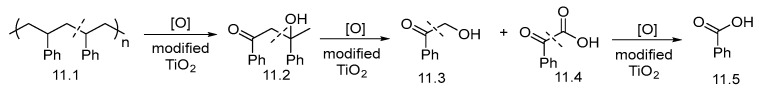
Mechanism of TiO_2_ catalyst degrading PS in oxygenated environment.

In 2023, Hongji Li and coworkers published a study detailing a photocatalytic degradation reaction using titanium (IV) oxide (TiO_2_) modified with surface functional groups as the photocatalyst and UV light at 370 nm as the irradiation source (Scheme 10) [37]. Benzoic acid was obtained as the primary product, with a 44.2% yield. TiO_2_ particles modified with alkaline molecules, potassium stearate (PSA), and *N,N*-diethyl-3-(trimethoxysilyl)-propan-1-amine (DTSPA) effectively photocatalyzed the PS degradation. The proposed mechanism for photocatalytic PS oxidation on organic-modified TiO_2_ (OMTiO_2_) involves enhanced charge separation due to organic modifiers under light irradiation (Scheme 11). Electrons reduce ground-state oxygen to superoxide radicals, which may further oxidize to singlet oxygen. The reaction initiates with C–H oxidation and C–C bond cleavage, facilitated by alkaline modifiers. Subsequent oxidation of carbonyl- and hydroxyl-containing intermediates leads to the formation of 2-hydroxyacetophenone 11.3, phenylglyoxylic acid 11.4, and ultimately benzoic acid 11.5.

The reaction conditions are mild, carried out at ambient temperature and atmospheric pressure, indicating favorable energy efficiency and operational simplicity. The PSA–TiO_2_ photocatalyst could be regenerated by immersion in THF and heating at 60 °C, while the DTSPA–TiO_2_ photocatalyst was regenerated by immersion in DCM. Various commercial PS products, including bottle caps, Styrofoam, disposable plastic cups, yogurt cups, and laboratory materials such as Petri dishes, were tested in the PSA–TiO_2_ system. The results showed successful degradation to benzoic acid for all samples except the yogurt cup, likely due to the presence of rutile in its composition. Although precise cost metrics were not reported, the use of recyclable photocatalysts and mild conditions suggests a promising path toward economically feasible and scalable PS degradation.

**Scheme 12:**
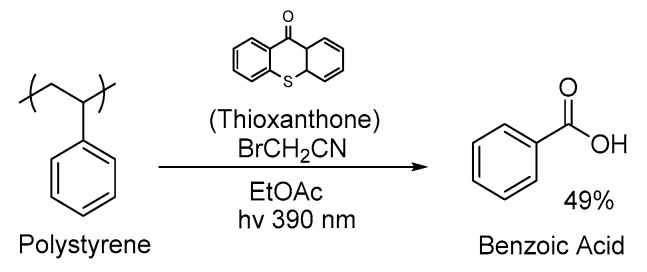
Photocatalytic degradation of PS via C via Synergistic Indirect HAT Catalysis.

**Scheme 13:**
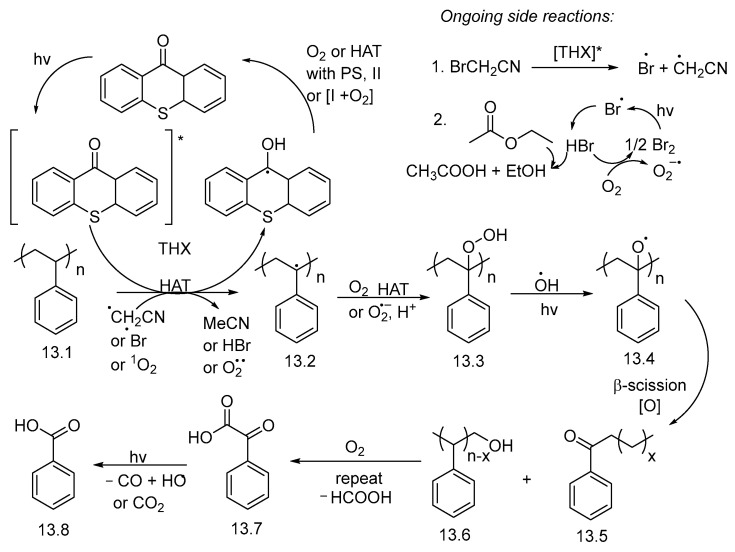
Mechanism of THX photocatalyst degrading PS via HAT in conjunction with bromoacetonitrile. * meant the excited state of the fluorenone.

In 2024, Christoforos G. Kokotos and coworkers published a study describing a photocatalytic degradation reaction for PS using BrCH_2_CN-thioxanthone (THX) as the photocatalyst and a UV light at 390 nm as the irradiation source (Scheme 12) [10]. Benzoic acid was yielded as the primary product at 49% yield. A proposed mechanism for the photochemical reaction is shown in Scheme 13. Upon exposure to UV light, THX is excited to its active state, enabling it to abstract a hydrogen atom from the PS backbone, forming radical 13.2. This PS radical then reacts with molecular oxygen, followed by a HAT to form peroxide 13.3. Upon further exposure to UV light in the presence of hydroxide (OH), the peroxide undergoes β-scission, resulting in the formation of two polymer chain fragments: 13.5 and 13.6. Through repeated HAT events and β-scission processes, phenylglyoxylic acid (13.7) is formed, along with some formic acid. Continued irradiation leads to the decarboxylation of phenylglyoxylic acid, ultimately yielding the final product, benzoic acid (13.8).

The reaction conditions are mild and easy to achieve, proceeding smoothly under ambient air without the need for an external oxygen source as the oxidant, thereby reducing complexity and resource requirements. The THX photocatalyst can be regenerated simply by exposure to oxygen, making it reusable for subsequent runs and helping to reduce both material use and overall costs. Various commercial PS products—including Styrofoam, disposable plastic cups of different colors, CD cases, ice cream foam containers, plastic spoons, knives, forks, and microwavable food containers—were tested. In all cases, successful degradation into benzoic acid was observed, with yields ranging from 24% to 46%. The method has also been demonstrated on a 20 g scale of PS, yielding benzoic acid at 45% yield, further supporting its scalability and potential for economically viable implementation in larger-scale settings.

**Scheme 14:**
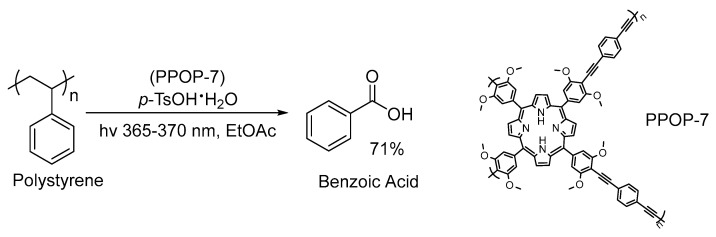
Photocatalytic degradation of PS using porous organic polymers (PPOPs).

**Scheme 15:**
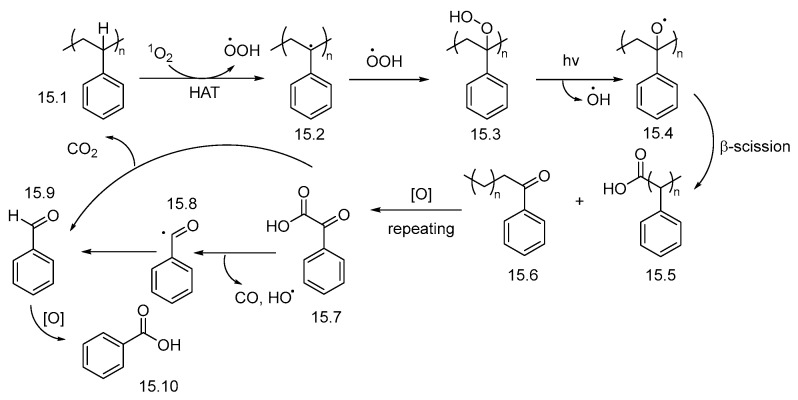
Mechanism of singlet oxygen degrading PS using porous organic polymers (PPOPs).

In 2023, Zheng and coworkers developed an aerobic photocatalytic degradation method for PS using porphyrin-based porous organic polymers (PPOPs) as photocatalysts and UV light in the 365–370 nm range (Scheme 14) [38]. Benzoic acid was obtained as the primary product with a 72% yield and excellent selectivity (97%). Among the PPOPs tested, PPOP-7, which contains a reduced tetraphenylporphyrin structure, exhibited the best performance. This is likely due to its large specific surface area (270.07 m^2^/g) and optimal pore size (2.6 nm). As with other HAT mechanisms (Scheme 15), a radical is generated on the PS backbone 15.2. Singlet oxygen, produced by PPOP-7 in the presence of molecular oxygen, serves as the HAT reagent. The resulting peroxy radical reacts with the benzyl radical (15.2) to form peroxide (15.3), which undergoes homolytic O–O bond cleavage, generating radical 15.4. Subsequent β-scission and repeated oxidation steps ultimately yield benzoic acid (15.10).

The reaction conditions are mild, carried out at ambient temperature and atmospheric pressure, which supports energy efficiency and operational simplicity. The PPOP-7 photocatalyst could be regenerated by exposure to UV light. Various commercial PS products, including Styrofoam, coffee cup lids, yogurt containers, plastic plates, food boxes, and SBS waste, were tested. The results showed successful degradation to benzoic acid, with yields ranging from 44% to 65%.

**Scheme 16:**
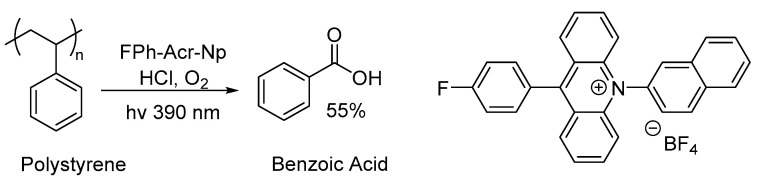
Photocatalytic degradation of PS via acridinium salt.

**Scheme 17:**
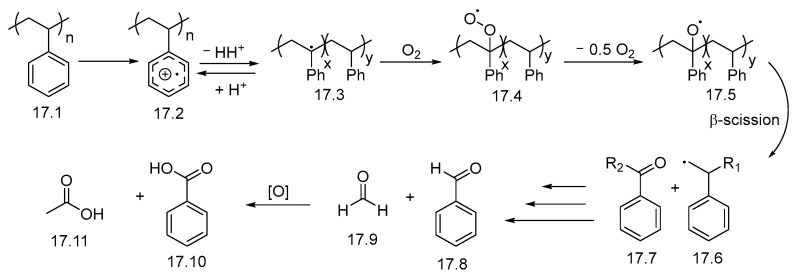
Mechanism of PS degradation using acridinium photocatalyst.

In 2023, Lim and coworkers reported the first photocatalytic degradation of PS using acridinium salts as the photocatalyst under 390 nm UV-light irradiation (Scheme 16) [39]. A non-intuitive fluorinated acridinium catalyst (FPh-Acr-Np) was identified as the optimal photocatalyst for converting PS to benzoic acid in useful yields (55%) at low catalyst loadings (5 mol%). It was hypothesized that acridinium salts could degrade PS via an oxidative mechanism, as these catalysts are capable of entering an excited, highly oxidative state upon light exposure. As shown in Scheme 17, PS undergoes oxidation by an excited acridinium salt catalyst, forming a benzylic radical intermediate (17.3), which is subsequently trapped by molecular oxygen to generate peroxyl radical 17.4. This intermediate then undergoes homolytic O–O bond cleavage, yielding radical 17.5, followed by β-scission. This process produces a primary radical chain end (17.6) and a phenyl ketone (17.7), both of which can further react, propagating additional chain cleavage. The degradation ultimately results in the formation of benzoic acid (17.10) and formic acid (17.11) as the terminal oxidation products.

The reaction conditions are mild, conducted at ambient temperature and atmospheric pressure, which enhances operational simplicity and energy efficiency. The FPh-Acr-Np photocatalyst was able to be regenerated by reacting with chloride ions or oxygen gas and then being exposed to UV light. Various commercial PS products, including Styrofoam, clear cup lids, black PS lids, and lab weighing boats, were tested, all showing successful degradation to benzoic acid, with yields ranging from 40% to 47%.

### 2.3. Broad-Spectrum Light Sources

**Scheme 18:**
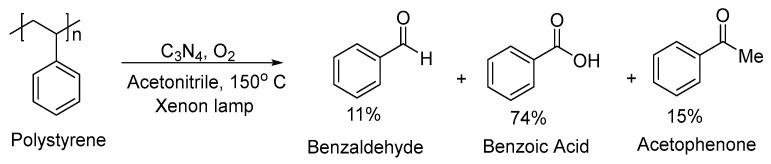
Photocatalytic degradation of PS using carbon nitride.

**Scheme 19:**
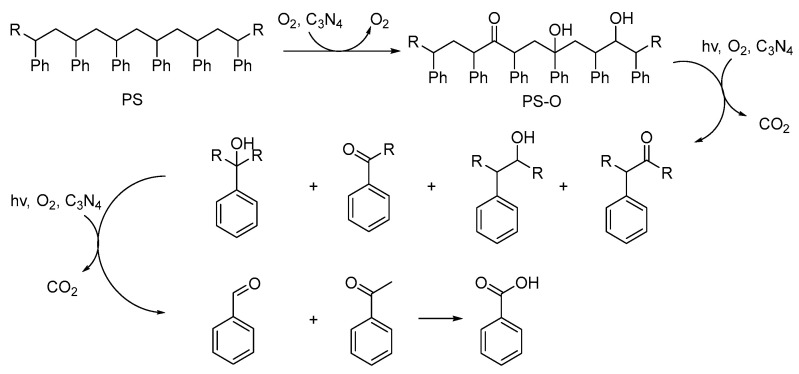
In-depth mechanisms of C-H oxidation and C-C scission.

In 2022, Cao and coworkers reported the photocatalytic degradation of PS utilizing a graphitic carbon nitride (C_3_N_4_) catalyst as the photocatalyst and a 300 W Xenon lamp as the light source (Scheme 18) [40]. The primary degradation products, benzoic acid, acetophenone, and benzaldehyde, were obtained in yields of 74%, 15%, and 11%, respectively. Unlike previous studies that focused on specific wavelengths of light, this work employed a broad-spectrum xenon lamp (280–800 nm). A semiconductor photocatalyst was selected due to its favorable properties, including high stability, tunable electronic structure, and strong light absorption. The authors proposed that in the presence of O_2_ gas and a heterogeneous photocatalyst, the C–H bonds in PS would be oxidized to form an activated intermediate (PS–O, Scheme 19). This would then undergo C–C bond cleavage followed by successive oxidation and β-scission steps, ultimately yielding benzaldehyde, acetophenone, benzoic acid, and CO_2_ as final products.

The reaction conditions were generally mild, as the process was conducted under ambient pressure without the use of toxic or expensive catalysts. However, moderately high temperatures (150 °C) were required to degrade PS, which introduces higher costs, necessitates temperature control, and poses potential safety risks. These factors could limit the economic feasibility and scalability of the method compared to other room-temperature approaches, though the simplicity of the catalyst system remains a practical advantage.

## 3. Non-Photocatalytic Upcycling

Non-photocatalytic upcycling differs from photocatalytic approaches in two key ways: it does not require light energy or any external light source, and it predominantly utilizes heterogeneous catalysts. Consequently, the energy needed to cleave carbon–carbon (C–C) bonds must come from another source, typically high temperatures. While effective, this reliance on thermal energy makes the process less energy-sustainable. The need for elevated temperatures not only increases energy consumption but also raises operational costs, demands more rigorous temperature control, and introduces greater safety concerns.

**Scheme 20:**
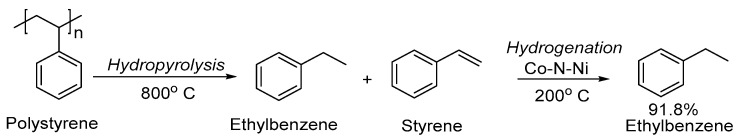
Degradation of PS via Thermochemical Hydrogenation.

In 2023, Runze Li and coworkers published a work involving non-photocatalytic degradation using a N-bridged Co, Ni dual-atom catalyst as a heterogeneous catalyst (Scheme 20) [41]. Ethylbenzene was obtained as the primary product with an impressive yield of 91.8%. This reaction is novel for its use of dual-atom catalysts (DACs) rather than single-atom catalysts (SACs). The rationale behind this choice lies in the inherent limitation of SACs: their isolated active sites can hinder catalytic pathways that require multiple coordinated active centers, thereby reducing catalytic efficiency. In contrast, DACs retain the cost-effectiveness associated with low metal loading while offering synergistic effects between the two active metal sites. These dual-atom sites can more effectively regulate the position of the d-band center and enhance electron/hole density through improved electron transfer.

However, the reaction conditions in this study were not particularly mild. Extremely high temperatures were required for both stages of the process, which increases operational costs, necessitates rigorous temperature control, and introduces safety risks. Notably, the DAC demonstrated excellent stability and recyclability—after nine regeneration cycles, no decline in catalytic activity was observed—which significantly reduced material cost per run. The method was also tested on commercially available polystyrene-based plastics, including Styrofoam, clear plastic cups, high-impact polystyrene (HIPS), and food containers. All showed successful degradation into ethylbenzene, with conversion rates exceeding 85%. In contrast, other polymers such as polyethylene terephthalate (PET), polyethylene (PE), and polypropylene (PP) exhibited negligible conversion, indicating strong selectivity for PS.

**Scheme 21:**
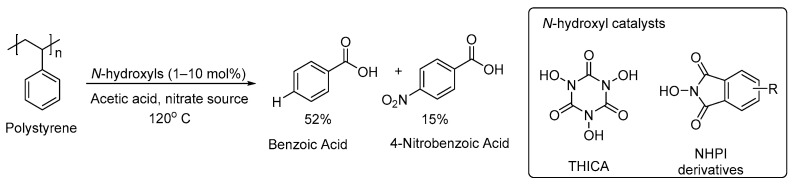
Non-photocatalytic degradation of PS via aerobic oxidative degradation of tertiary C-H bond.

**Scheme 22:**
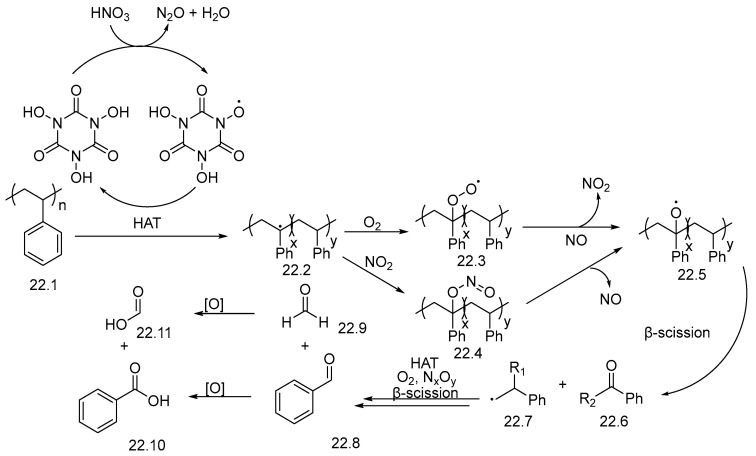
Mechanism of THICA catalyst catalyzing PS degradation.

In 2023, Ong and coworkers published a study utilizing *N*-hydroxyl catalysts to oxidatively degrade PS (Scheme 21) [42]. Benzoic acid and 4-nitrobenzoic acid were identified as the primary products. The formation of 4-nitrobenzoic acid is attributed to nitration of the aromatic rings of PS by HNO_3_, occurring prior to chain cleavage. This method differs from previously discussed approaches in that it does not employ metal catalysts or conventional photoredox catalysis. Instead, it relies on organocatalysis, a relatively limited catalytic strategy that has primarily been applied to polymers with inherent cleavage points, such as polyesters [43,44]. N-hydroxyl catalysts such as THICA (N, N′, N″-trihydroxyisocyanuric acid) and NHPIs (N-hydroxy phthalimide derivatives) were utilized. Similarly to the HAT reagents described in earlier studies, these *N*-hydroxyl catalysts facilitated the oxidation of PS via its tertiary C–H bonds in the presence of a nitrate source (nitric acid) [45,46]. As illustrated in Scheme 22, the reaction proceeds through the formation of radical intermediate 22.2, which is trapped by molecular oxygen to yield peroxyl radical 22.3. This peroxyl radical reacts with nitric oxide (NO), undergoing homolytic O–O bond cleavage to generate nitrogen dioxide (NO_2_) and oxo-radical 22.5. This leads to subsequent C–C bond scissions and further oxidation, ultimately forming the high-value product benzoic acid (22.10).

The reaction conditions were generally mild, as the experiment was conducted at ambient pressure and did not require expensive or toxic reagents. However, elevated temperatures were necessary to facilitate the process, which may increase energy costs and introduce some limitations for large-scale implementation. This method was tested on a variety of PS products, including Styrofoam food containers, black and clear plastic cup lids, a weighing boat, and PS beads. Successful degradation was observed across all five substrates, including the black plastic lid, which is notable because such colored plastics are typically resistant to photoredox-catalyzed degradation due to their light-absorbing additives. This demonstrates the potential of this organocatalytic method as a viable alternative to photoredox-based approaches. Moreover, the method has also shown the ability to degrade other plastics, such as poly(4-methylstyrene), leading to the formation of new products like terephthalic acid. The scalability of this approach has been demonstrated through successful degradation of up to 10.4 g of pure PS, indicating potential for future development toward economically viable larger-scale applications

**Scheme 23:**
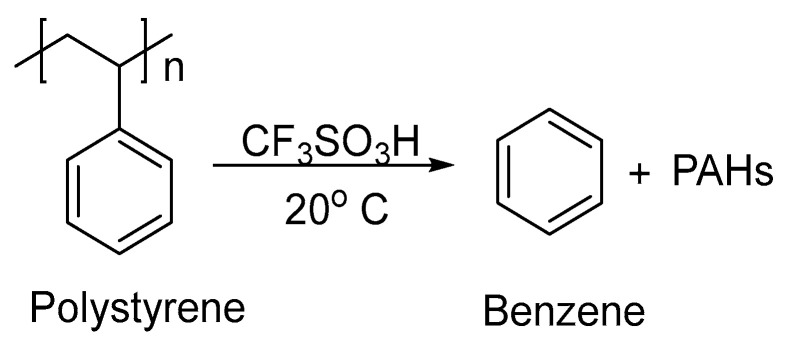
Non-photocatalytic degradation of PS via cross-linking and subsequent degradation of cross-links.

**Scheme 24:**
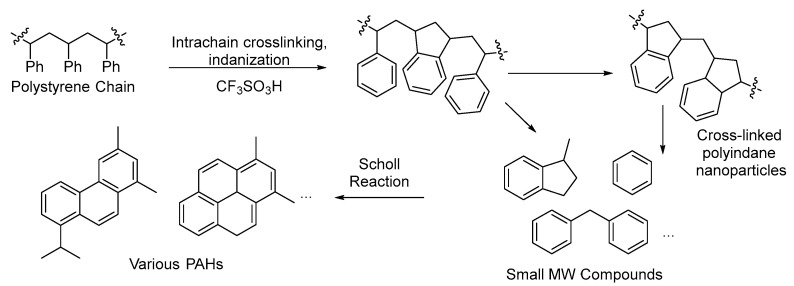
In-depth mechanism of cross-linking and degradation of cross-linking.

In 2023, Thigale and coworkers published a study involving the use of triflic acid to degrade PS, producing various polycyclic aromatic hydrocarbons (PAHs) at 20 °C (Scheme 23) [47]. This method differs from all other approaches discussed, as it does not utilize a hydrogen atom transfer (HAT) process or generate any radicals to break the carbon backbone of PS. As shown in Scheme 24, the degradation of PS by triflic acid first involves intrachain cross-linking, which leads to the formation of polymer nanoparticles and the simultaneous transformation of PS chains into poly(styrene-co-indan). This is followed by the degradation of the cross-linked polymer nanoparticles into low-molecular-weight compounds. Finally, a Scholl reaction occurs, resulting in the formation of various PAHs.

## 4. Conclusions

Both photocatalytic and non-photocatalytic approaches offer promising pathways for the upcycling of PS waste into valuable chemical compounds. Photocatalytic upcycling primarily leverages renewable and cost-effective sources of energy, such as UV or visible light, to generate reactive radical species, enabling efficient degradation of PS under mild and relatively safe reaction conditions. In contrast, non-photocatalytic methods typically rely on high-temperature processes and heterogeneous catalysts, which pose higher energy demands, increased operational costs, and potential safety hazards associated with heat management.

A common mechanistic feature observed in various PS degradation strategies is the hydrogen atom transfer (HAT) process, where radicals abstract hydrogen atoms from the saturated carbon backbone of PS, facilitating subsequent backbone cleavage. While photocatalytic methods generally align more closely with sustainability goals due to their mild conditions and environmental friendliness, they often suffer from comparatively moderate yields, frequently below 50%. On the other hand, although non-photocatalytic approaches may achieve higher yields for certain products, these processes usually come with higher environmental and safety concerns due to extreme reaction conditions.

A comparative assessment of degradability across the reviewed methods reveals several key trends. Photocatalytic strategies generally enable degradation under ambient temperature and pressure, offering yields of benzoic acid typically ranging from 40% to 65% depending on catalyst design, light source, and substrate type. These methods perform well on a wide range of commercial PS products, although colored plastics, such as black lids, often exhibit reduced conversion due to limited light penetration. Organocatalytic methods show broader substrate tolerance and competitive yields but may require elevated temperatures. Non-photocatalytic thermal methods, while capable of achieving high conversion rates, typically demand harsh conditions and are more limited in polymer scope. Overall, degradability is strongly influenced by catalyst reusability, energy input, and compatibility with mixed or contaminated waste streams, all of which play crucial roles in assessing the practical viability of each method.

Despite these advancements, key challenges remain for the broader implementation of PS upcycling. Photocatalytic methods often face limited light penetration in colored plastics, moderate yields, and issues with catalyst stability. Non-photocatalytic approaches typically require high temperatures and oxidants, increasing energy costs and safety concerns. Both methods must also contend with contaminants and additives in real-world PS waste, which can reduce efficiency and product purity. Addressing these limitations is crucial for the development of scalable, cost-effective, and sustainable solutions.

Given the continued and projected increase in global plastic pollution, including PS waste, it is essential to advance both types of methods. Ongoing research should focus on enhancing yields, optimizing reaction conditions, exploring catalyst recyclability, and scaling up processes for practical industrial applications. By systematically improving these upcycling technologies, significant progress can be made toward effectively addressing the mounting PS waste challenge and moving closer to a sustainable and circular plastic economy.
